# Interaction of ACTN3 gene polymorphism and muscle imbalance effects on kinematic efficiency in combat sports athletes

**DOI:** 10.20463/jenb.2016.06.20.2.1

**Published:** 2016-06-30

**Authors:** Hansang Jung, Namju Lee, Sok Park

**Affiliations:** 1 College of Sports Science, Kangnam University, Yongin Republic of Korea; 2 Department of Sports Health Medicine, School of Sports Science, Jungwon University, Goesan Republic of Korea; 3 Department of Sports Leadership, College of Natural Science, Kwangwoon University, Seoul Republic of Korea

**Keywords:** ACTN3, Muscle imbalance, Athletes

## Abstract

**[Purpose]:**

The purpose of this study was to determine the interaction of ACTN3 gene polymorphism and muscle imbalance effects on kinematic efficiency changes in combat sports athletes.

**[Methods]:**

Six types of combat sports athletes (Judo, Taekwondo, boxing, kendo, wrestling, and Korean Ssi-reum) participated in the study. ATCN3 gene polymorphism and muscle imbalance in lower extremity were evaluated followed by analysis of differences of moment in hip, knee, and ankle joint during V-cut jumping and stop. To examine the moment difference due to an interaction of ATCN3 polymorphism and muscle imbalance, all participants were divided into 4 groups (R+MB, R+MIB, X+MB, and X+MIB).

**[Results]:**

There was no significant difference of hip, knee, and ankle joint moment in R allele and X allele during V-cut jumping and stop based on ACTN3 gene polymorphism. Otherwise, muscle imbalance of knee moment in X-axis and ground reaction force of knee in Z-axis showed a higher significance in muscle imbalance during V-cut jumping and stop compared to muscle balance (p<0.05). In addition, joint analysis showed that muscle imbalance in X allele group had significantly higher knee moment of V-cut ground reaction force in X-axis and higher ankle moment of jumping ground reaction force in X and Z-axis compared to muscle balance with R and/or X group (p <0.05).

**[Conclusion]:**

This study confirmed that muscle imbalance in lower extremity of combat athletes might induce higher risk factors of sports injury incidence than genetic factor and training might reduce the ratio of sports injury risk incidence.

## NTRODUCTION

Combat as a type of contact sport in athletes should mainly consider connecting movement skills for quick technique due to its characteristics, which results in a higher expected risk of sports injury especially to joint areas^1,2^. Moreover, rapid agility requires efficient muscle control and regulation during muscle contraction; and mostly, lower extremity efficacy is known to be caused by the balance of flexors and extensors of the thigh area in the human body^3,4^. However, the balance of hamstrings to quadriceps ratio (H/Q) is usually dependent on the distinct characteristics of muscle fibers and therefore, understandings of the muscle fiber characters related to the gene of the individuals is required^5,6^.

Recently, 52 genotypes that highly contribute to functions for best performance in elite athletes are reported and gene polymorphism mainly controls the sensitivity difference of the individuals due to human body structure and function^7^. Moreover, this phenomenon induces latent response of the genetic factors following training and leads human body changes and development at the molecular level^8^. ACTN3 is considered as one of the most important genes to be highly related to physical fitness (aerobic and anaerobic) and directly contributes to the structure and function of the muscle fibers^9^. ACTN3 is uniquely and restrictively expressed within the fast twitch fibers, which is related to muscular strength and agility in athletes^10^. Recent review studies suggest that ATCN3 expression in fast twitch fiber is a positive aspect and has a higher relationship of ultrastructural muscle damage such as z-line streaming in the muscle fibers due to eccentric contraction under long term and higher intensity situation as a negative aspect. Therefore, studies are required to address these opposing characteristics of ACTN3 to quantify its exact function^9-12^. Previous studies reported that ATCN3 greatly contributes to ACTN3 R allele for quickening muscle contraction speed and this might affect performance capacity enhancement and physical fitness in elite athletes; however, ACTN3 R allele is expected to have a negative effect on decelerating movement with fast acceleration of the muscle contraction such as a quick direction change and control. Lower extremity joint efficacy induced by muscle imbalance, a known injury incidence factor, has not been fully discussed previously. An ATCN3 study of Korean athletes reported 48% of the R allele and 52% of the X allele in combat sports athletes unlike non-athletic population (R allele: 33%, X allele: 67%), sprinters (R allele: 32%, X allele: 68%), and endurance athletes (R allele: 28%, X allele: 72%) and otherwise, similar to middle distance race runners (R allele: 48%, X allele: 52%)^13^. Middle distance runners reportedly have a higher injury incidence rate of the lower extremity joint during training, as compared to sprinters and endurance athletes^14^ and the characteristics of the muscle fibers is the expected cause of injury incidence^15^. However, athletic injury due to the characteristics of muscle fibers is too complex to discuss as an etiological factor. Therefore, the purpose of this study was to determine the interaction of ACTN3 gene polymorphism and muscle imbalance effects on kinematic efficiency changes focusing on lower extremity in combat sports athletes.

## METHODS

### Participants

**Figure 1.**
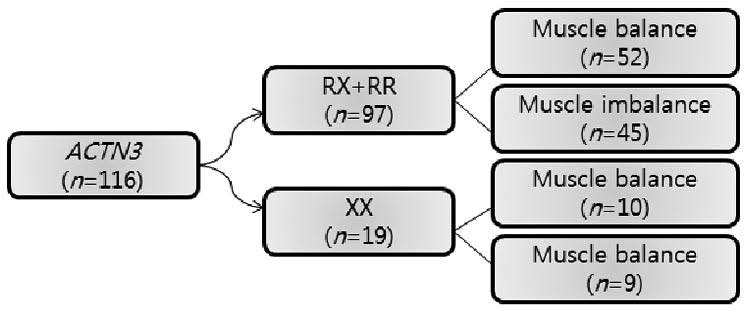
Study design

One hundred and sixteen combat sports athletes from six types of combat sports participated in the study as shown in Table 1. Total calculation of these study participants was according to previous ACTN3 character reports 9, 11, 16 and was calculated by 95% of the confidence interval with 2.5% sampling error (Table 1). This study design was a randomized block design and quantified musculoskeletal efficacy difference in the lower extremity among athletes based upon ACTN3 polymorphism (R allele and X allele) (Figure 1).

**Table 1.** Sports types of the study participants

Sports Types	Persons	%
Judo	25	21.6
Taekwondo	40	34.5
Boxing	13	11.2
Kendo	12	10.3
Wrestling	13	11.2
Korean Ssi-reum	13	11.2

### Blood and gDNA sampling

In all participants, 5mL blood was collected from the mid brachial vein after 12 hours fasting. To analyze gene polymorphism, genomic DNA sampling was conducted by using Gentra® Puregene® Blood Kit (Cat no. 158467, QIAGEN, USA) and all procedures followed the recommended methods. Extracted genomic DNA was stored at 4°C.

### ACTN3 R577X polymorphism analysis

To analyze ACTN3 R577X (rs1815739) polymorphism from the extracted gDNA, MGB TaqMan® SNP genotyping assay method was used in this study. The gene-specific primer was obtained from ACTN3 sequence listing of the Gene Bank at ABI (Applied Biosystems Incooperate, USA) and produced by TaqMan probes that can recognize R allele and X allele including SNP. Sequence listings were as follows; Forward: 5’ACGATC-AGT-TCA-AGG-CAAGGC- AAC-ACT-3’, Reverse: 5’-ACC-CTG-GAT-GCCCAT- GAT-G-3’, VIC (R-allele specificprobe): 5’-TCGCTC- TCG -GTC-CCA-TGA-TG-3’MGB, FAM(X-allele specific probe): 5’-TCG-CTC-TCG-GTC-AGC- 3’MGB. Gene amplification was conducted with cycling conditions of 15 second denaturation at 92°C and with 1-minute annealing and extension at 57°C for 40 cycles with a reaction mixture of gDNA 10~50 ng, 150 pmol primer, 70 nM VIC probe, and 70 Nm FAM probe by using TaqMan Genotyping Assasy Master Mix and ABI 7900HT (Applied Biosystems Incooperate, USA).

### Isokinetic angular measurement to estimate muscle imbalance

Thigh muscle balance test was conducted by isokinetic equipment (Isomed 2000, Germany) focusing on flexion and extension exercise in the knee joint. Before measurement, all participants practiced the isokinetic equipment at 50-70% of maximal intensity with 5-10 times and negative stimulation was used for maximizing muscular revelation^17^.

Dynamometer laser to perform maximal exercise was used and fixed at the mid knee joint with 85° back rest and the seat was adjusted by each participant’s lower extremity length. Range of motion was set at 110° based upon anatomical neutral position. During flexion exercise, automatic program of the machine controlled lower extremity weight calibration and seat and the shoulder belt was identically applied to all participants. Measurement load velocity was set at 60°/sec and both legs were measured (left and right leg order). Peak torque of extension and flexion strength was measured to estimate muscle balance. All participants were divided into normal and abnormal H/Q ratio group based on the recommended H/Q ratio of 60%^18,19^.

### Lower extremity muscle efficacy test

Before measurement, all participants were fully informed of the study purpose and experimental procedures. To prevent injury and obtain best quality data, each participant conducted warming up. To set up the global reference frame, L-Frame was used to constitute coordinate calculation for X, Y, and Z axis. X axis stands for left and right direction, Y axis stands for front and rear direction, and Z axis stands for upper and lower direction in the coordinates. Segment length and diameter of each participant including ASIS (anterior superior iliac spine) was measured with a tape measure (Lufkin W606PM flexible steel tape, USA) and an anthropometer (GPM, Swiss). Twenty- one outside reflection markers (15mm) were attached at right/left side of a acromion processes, anterior superior iliac spines, sacrum (posterior superior iliac spine 1/2), great trochanters, lateral aspect right middle of thighs and shanks, medial and lateral femoral condyles, malleoli, and second metatarsal heads. Using outside reflection markers, hip, knee, and ankle joint was determined under midpoint method20 and then body segment coordinates were set. Human trunk local coordinates were determined by Z as a major axis, X as a vector from the left shoulder mark connected to the right shoulder mark, and Y as a vector based on rectangular coordinates from X and Z axis.

**Figure 2.**
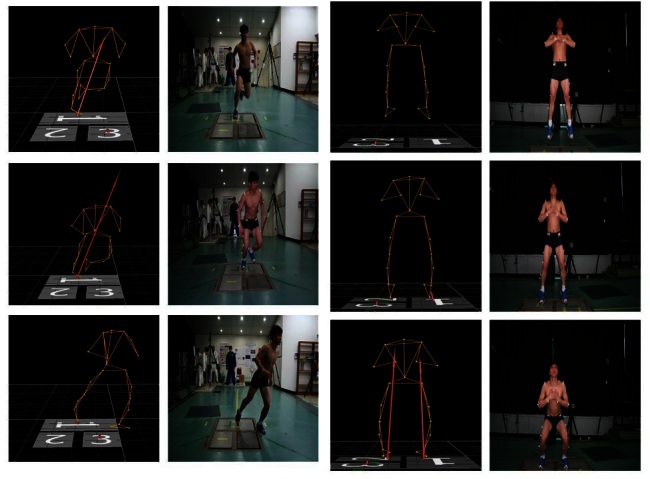
V-cut and jumping and stopping

In this study, 2 types of decelerate movement as a V-cut and jumping and stopping (Figure 2) were conducted. V-cut movement consisted of straight line running, stepping on the ground reaction force (GRF) plate, and a 45-degree quick turn to the opposite direction, in order. All participants conducted both leg V-cut movement. Jumping and stopping consisted of straight line running, jumping on the GRF plate, and then both leg landing on the GRF plate. Straight line running in this study speed was set at 3.5 ± 0.2 m/s and all participants’ running speed was controlled by 2 photoelectric sensors (Photoelectric sensor, Visol Inc., Gwangmyeong, Korea). We conducted five repeated measurements of decelerate movement until obtaining optimal data for each participant. To prevent injury and fatigue, all participants wore running shoes and took 5-minute break time.

### Data Analysis

SPSS 16.0 for Window (Chicago, IL, USA) was used for analyses and statistical significance was set at p<.05. Descriptive statistics included mean and the standard deviation of all variables. To quantify this study purpose, a cross tab analysis was used by using the cut-off point (H/Q ratio as 60%) and dividing groups into abnormal as 1 and normal as 0 based on R allele and T allele. Kinematic factor difference based upon complexion function of genetic traits in this study was quantified by using one-way ANOVA.

## RESULTS

Among the study participants (n=119), 94 were R allele and 19 were X allele. Age, height, weight, and body fat showed no significant difference based on ACTN3 gene polymorphism (Table 2). Also, there was no significant difference of R allele and X allele in muscle imbalance trend based on ACTN3 gene polymorphism (Table 3). Kinematic efficacy difference based on ACTN3 gene polymorphism indicated lower mean values of knee moment of X, Y, and Z in X allele group during V-cut compared to R allele group; otherwise, knee moment of X, Y, and Z in X allele group showed higher mean values during jumping compared to R allele group. However, there was no significant difference of knee and ankle joint moment in both groups.

**Table 2.** Physical characteristics of ACTN3 gene polymorphism

Gene Polymorphism	Age, years	Height, cm	Weight, kg	Body fat, kg
RR+RX, n=94	19.60±1.20	175.44±6.19	74.46±12.42	10.48±6.7
XX, n=19	19.79±1.32	174.18±6.35	74.07±10.47	10.85±5.7
p for trend	0.53	0.42	0.90	0.82

**Table 3.** Muscle imbalance trend analysis based on ACTN3 gene polymorphism

	Muscle balance (n=62)	Muscle imbalance (n=54)	χ^2^	P
RR+RX(n=94)	52(53.6%)	45(46.4%)	0.006	0.94
XX(n=19)	10(52.6%)	9(47.4%)		

**Figure 3.**
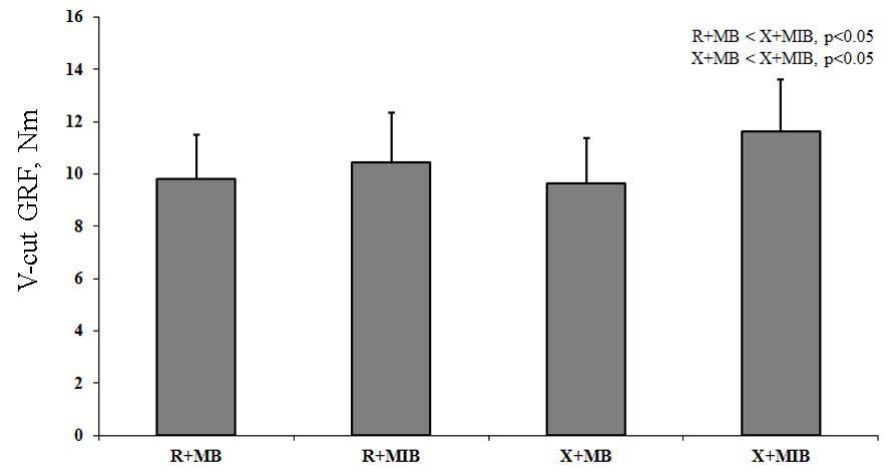
V-cut ground reaction force (GRF) based on ACTN3 gene polymorphism and muscle balance (R; R allele , X; X allele, MB; muscle balance, MIB; muscle imbalance)

**Figure 4.**
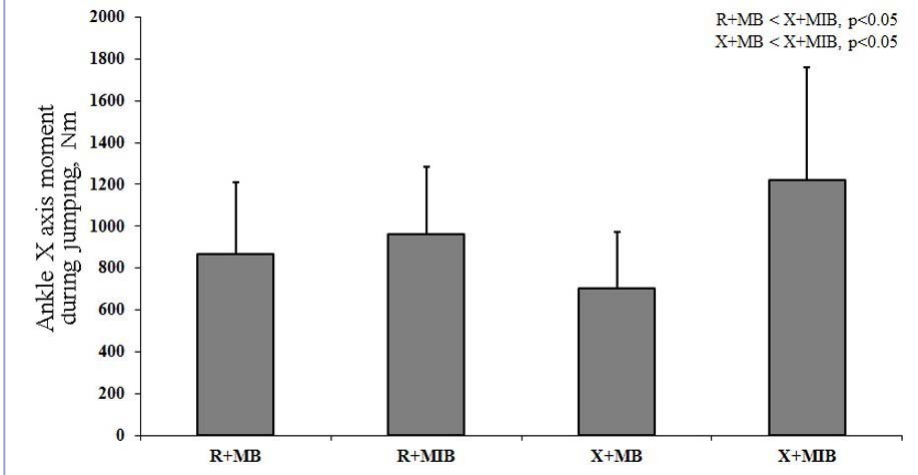
Ankle X axis moment based on an interaction of ACTN3 gene polymorphism and muscle imbalance during jumping (R; R allele , X; X allele,, MB; muscle balance, MIB; muscle imbalance)

**Figure 5.**
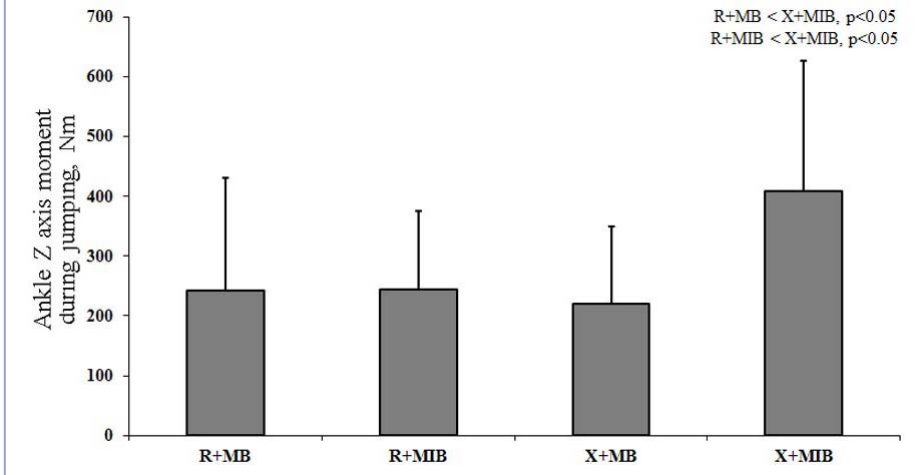
Ankle Z axis moment based on an interaction of ACTN3 gene polymorphism and muscle imbalance during jumping (R; R allele, X; X allele, MB; muscle balance, MIB; muscle imbalance)

**Figure 6.**
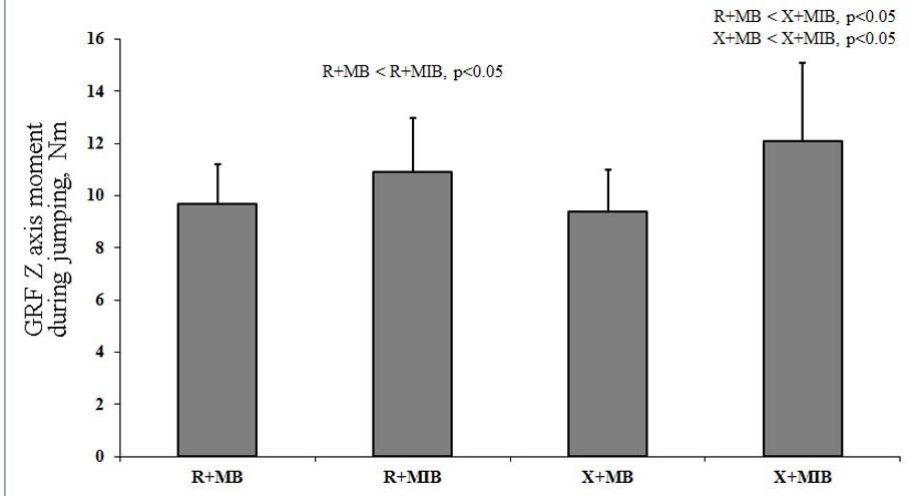
GRF Z axis moment based on an interaction of ACTN3 gene polymorphism and muscle imbalance during jumping (R; R allele, X; X allele, MB; muscle balance, MIB; muscle imbalance, GRF: ground reaction force)

X allele group showed significant difference in GRF Y axis during V-cut and jumping, as compared to R allele group (Table 4). Kinematic efficacy difference based on muscle balance showed a significantly higher value of knee X axis moment and GRF Z axis during V-cut in X allele group, as compared to R allele group (p<0.05). Additionally, kinematic efficacy difference based on muscle balance indicated a significantly higher knee X axis moment, ankle X moment, and GRF Z axis during jumping in X allele group compared to R allele group (p<0.05) (Table 5). During V-cut, kinematic efficacy based on an interaction of ACTN3 gene polymorphism and muscle imbalance showed that GRF Z axis was significantly higher in X allele with muscle imbalance group, as compared to R allele and X allele with muscle balance group (p<0.05) (Figure 3). During jumping, kinematic efficacy based on an interaction of ACTN3 gene polymorphism and muscle imbalance showed that X axis moment was significantly higher in X allele with muscle imbalance group, as compared to R allele and X allele with muscle balance group (p<0.05) (Figure 4). During jumping, kinematic efficacy based on an interaction of ACTN3 gene polymorphism and muscle imbalance showed that Z axis moment was significantly higher in X allele with muscle imbalance group, as compared to R allele with muscle balance and imbalance group (p<0.05) (Figure 5). During jumping, kinematic efficacy based on an interaction of ACTN3 gene polymorphism and muscle imbalance showed that Z axis moment was significantly higher in X allele with muscle imbalance group, as compared to R allele and X allele with muscle balance group (p<0.05); and especially, Z axis moment was significantly higher in R allele with muscle imbalance group, as compared to R allele with muscle balance group (p<0.05) (Figure 6).

**Table 4.** Kinematic efficacy difference based on ACTN3 gene polymorphism

	Variables	RR+RX	XX	P
V-cut, Nm	Knee X axis moment	1145.59±507.68	1050.16±581.44	0.47
	Knee Y axis moment	65.97±339.82	43.90±304.17	0.79
	Knee Z axis moment	52.11±85.55	31.19±107.04	0.35
	Ankle X axis moment	997.13±415.59	1144.95±505.20	0.17
	Ankle Y axis moment	-2.71±134.37	-12.47±143.02	0.78
	Ankle Z axis moment	176.94±131.04	146.68±111.06	0.35
	Pelvis X axis moment	1467.54±406.56	1633.32±578.37	0.13
	Pelvis Y axis moment	-662.25±505.86	-648.14±644.72	0.92
	Pelvis Z axis moment	236.06±194.41	193.08±190.21	0.38
	GRF X axis	-0.16±0.34	-0.25±0.32	0.34
	GRF Y axis	0.81±0.61	1.12±0.69	0.05
	GRF Z axis	10.13±1.86	10.59±2.06	0.33
Jumping, Nm	Knee X axis moment	1219.03±519.59	1293.11±513.23	0.57
	Knee Y axis moment	175.24±372.59	266.46±324.35	0.32
	Knee Z axis moment	244.06±96.46	261.42±91.47	0.47
	Ankle X axis moment	911.02±336.52	949.16±487.99	0.68
	Ankle Y axis moment	220.15±93.78	210.21±86.57	0.67
	Ankle Z axis moment	243.54±164.40	310.34±197.44	0.12
	Pelvis X axis moment	1527.20±411.47	1509.47±582.95	0.87
	Pelvis Y axis moment	-459.97±511.09	-421.93±519.24	0.77
	Pelvis Z axis moment	68.02±189.24	35.03±177.39	0.48
	GRF X axis	-0.22±0.36	-0.23±0.39	0.94
	GRF Y axis	-0.79±0.62	-1.09±0.71	0.06
	GRF Z axis	10.34±1.94	10.74±2.69	0.44

All data were expressed as the mean and the standard deviation.

GRF: ground reaction force

**Table 5.** Kinematic efficacy difference based on muscle balance

	Variables	RR+RX	XX	P
V-cut, Nm	Knee X axis moment	1038.91±424.48	1234.50±596.80	**0.04**
	Knee Y axis moment	48.30±303.94	78.50±365.94	0.62
	Knee Z axis moment	62.01±78.82	33.40±98.42	0.09
	Ankle X axis moment	984.83±469.61	1063.25±385.88	0.33
	Ankle Y axis moment	8.05±130.08	-18.49±140.79	0.29
	Ankle Z axis moment	166.72±131.29	178.02±125.13	0.64
	Pelvis X axis moment	1513.55±463.33	1473.04±416.27	0.62
	Pelvis Y axis moment	-658.00±464.48	-662.16±597.03	0.96
	Pelvis Z axis moment	220.75±185.08	238.50±204.21	0.63
	GRF X axis	-0.18±0.31	-0.17±0.37	0.89
	GRF Y axis	0.80±0.63	0.92±0.64	0.37
	GRF Z axis	9.82±1.69	10.64±2.02	**0.02**
Jumping, Nm	Knee X axis moment	1113.35±393.77	1366.43±605.62	**0.008**
	Knee Y axis moment	162.50±351.43	221.97±381.57	0.38
	Knee Z axis moment	249.34±91.09	244.11±101.09	0.77
	Ankle X axis moment	839.74±338.81	1006.29±373.11	**0.01**
	Ankle Y axis moment	212.30±81.03	225.65±104.17	0.44
	Ankle Z axis moment	239.43±180.41	271.76±159.70	0.31
	Pelvis X axis moment	1471.92±421.99	1584.43±458.62	0.17
	Pelvis Y axis moment	-500.29±438.32	-400.29±581.87	0.29
	Pelvis Z axis moment	80.09±179.42	42.60±195.09	0.28
	GRF X axis	-0.23±0.34	-0.22±0.40	0.94
	GRF Y axis	-0.78±0.64	-0.91±0.65	0.26
	GRF Z axis	9.73±1.56	11.17±2.32	**<0.0001**

All data were expressed as the mean and the standard deviation.

GRF: ground reaction force

## DISCUSSION

The study results confirmed an association in kinematic efficacy and muscle balance of H/Q ratio based on ACTN3 gene polymorphism in fast twitch muscle fibers. Combat sports athletes are expected to have a higher incidence of quick direction change with agility and therefore, they may have a higher injury risk according to H/Q ratio imbalance during decelerating movement, as compared to sprinters and endurance sports athletes^14,15^. In this study, knee X axis moment and GRF Z axis with muscle imbalance group showed significantly higher values, as compared to muscle balance group during V-cut test, confirming that combat sports athletes with muscle imbalance may have a higher resistant load on the knee joint due to the rapid direction change, as compared to muscle balance group. In particular, GRF Z axis group difference during V-cut test showed that muscle imbalance group received increased load on the knee joint, as compared to muscle balance group. This might evoke distributed load on the right and left side of the lower extremity, thus increasing overall load on the lower extremity. This result implies that agonist in the lower extremity does not efficiently decrease the speed of the antagonist’s decelerating mechanism during decelerating movement in the lower extremity, which may increase injury incidence of the knee joint^21^.

During agility movement, the extent of ACTN3 contribution to fast twitch fiber contraction is unclear; however, z-line streaming damage is expected during repetitive and long term muscle contraction such as an eccentric contraction^9,11,12^. Contrary to expectation, we found no significant difference of kinematic efficacy in the lower extremity based on ACTN3 gene polymorphism during rapid agility movement and vertical jumping; in addition, ACTN3 contributed during deceleration.

This study confirmed a significantly higher value of GRF during V-cut and jumping, ankle X axis moment during jumping, ankle Z axis moment during jumping, and GRF Z axis moment during jumping based on the interaction of the knee muscle imbalance and ACTN3 gene polymorphism in X allele group with knee muscle imbalance, as compared to R allele group with muscle imbalance and X allele group with muscle balance. Although these results did not show identical trends, on an average, X allele with knee muscle imbalance group showed higher risk of injury risk during agility and strength movement in combat sports athletes. Despite no similar previous studies that clearly explain the exact mechanism, it is likely that an interaction of ACTN3 gene polymorphism and muscle balance during agility and strength movement did not efficiently contribute to decreased risk of lower extremity injury incidence. Further studies are needed to determine the exact contribution ratio on evoking muscle power by using EMG under longitudinal study setting.

## CONCLUSION

Although ACTN3 gene polymorphism did not directly increase the lower extremity injury incidence in combat sports athletes, knee muscle imbalance of combat sports athletes might increase knee joint injury incidence; in addition, there is an association with muscular imbalance and knee and ankle injury incidence risk during rapid agility movement. Therefore, this study confirmed that muscle imbalance in lower extremity of combat athletes might induce higher risk factors of sports injury incidence, as compared to genetic factor and also confirmed that training might reduce the ratio of sports injury risk incidence in combat athletes.
